# High-Resolution Analysis Identifies High Frequency of *KIR-A* Haplotypes and Inhibitory Interactions of KIR With HLA Class I in Zhejiang Han

**DOI:** 10.3389/fimmu.2021.640334

**Published:** 2021-04-30

**Authors:** Sudan Tao, Yanmin He, Katherine M. Kichula, Jielin Wang, Ji He, Paul J. Norman, Faming Zhu

**Affiliations:** ^1^ Blood Center of Zhejiang Province, Key Laboratory of Blood Safety Research of Zhejiang Province, Hangzhou, China; ^2^ Division of Biomedical Informatics and Personalized Medicine, and Department of Immunology and Microbiology, University of Colorado Anschutz Medical Campus, Aurora, CO, United States

**Keywords:** KIR, HLA, KIR/HLA interaction, Zhejiang Han population, NK cells

## Abstract

Killer cell immunoglobulin-like receptors (KIR) interact with human leukocyte antigen (HLA) class I molecules, modulating critical NK cell functions in the maintenance of human health. Characterizing the distribution and characteristics of KIR and HLA allotype diversity across defined human populations is thus essential for understanding the multiple associations with disease, and for directing therapies. In this study of 176 Zhejiang Han individuals from Southeastern China, we describe diversity of the highly polymorphic *KIR* and *HLA class I* genes at high resolution. *KIR-A* haplotypes, which carry four inhibitory receptors specific for HLA-A, B or C, are known to associate with protection from infection and some cancers. We show the Chinese Southern Han from Zhejiang are characterized by a high frequency of *KIR-A* haplotypes and a high frequency of C1 KIR ligands. Accordingly, interactions of inhibitory KIR2DL3 with C1^+^HLA are more frequent in Zhejiang Han than populations outside East Asia. Zhejiang Han exhibit greater diversity of inhibitory than activating KIR, with three-domain inhibitory KIR exhibiting the greatest degree of polymorphism. As distinguished by gene copy number and allele content, 54 centromeric and 37 telomeric haplotypes were observed. We observed 6% of the population to have *KIR* haplotypes containing large-scale duplications or deletions that include complete genes. A unique truncated haplotype containing only *KIR2DL4* in the telomeric region was also identified. An additional feature is the high frequency of HLA-B*46:01, which may have arisen due to selection pressure from infectious disease. This study will provide further insight into the role of *KIR* and *HLA* polymorphism in disease susceptibility of Zhejiang Chinese.

## Introduction

Natural killer (NK) cells are innate immune effectors having multiple important roles in human health. NK cells are critical in controlling the outcome of infections, tumors and fetal implantation, as well as organ transplantation and immunotherapies (1–5). In these roles, NK cell functions are modulated by interactions of their cell surface receptors with ligands expressed by tissue cells (6, 7). In humans, the interactions include polymorphic combinations of killer cell immunoglobulin-like receptors (KIR) with human leukocyte antigen (HLA) class I ligands (8, 9). The extreme genetic variation of KIR and HLA class I directly impacts NK cell functions, and associates with multiple immune-mediated diseases (10, 11). Also directly affected by combinatorial diversity, and influencing effector functions in health and disease, maturation of NK cells is guided through appropriate interactions of KIR with HLA class I (12–14).

The *KIR* locus of chromosome 19 (19q13.4) comprises up to 13 genes encoding receptors having distinct ligand specificities and inhibitory or activating functions (15, 16). Encoded by each *KIR* haplotype are from one to four inhibitory receptors and up to five activating receptors specific for HLA-A, B or C. Four framework genes, present in most individuals, form the basic structure of the locus. Their arrangement divides the *KIR* locus into centromeric (*KIR3DL3* to *KIR3DP1*) and telomeric (*KIR2DL4* to *KIR3DL2*) oriented regions. Structural diversity of the *KIR* locus is formed through recombination events that duplicate or delete entire genes, or deletions that create in-frame gene fusions (17, 18). This genetic variation thus has direct consequences for NK cell functions, which are further diversified by the alleles of KIR and their HLA class I ligands (19, 20). Within HLA-A, B and C, only those allotypes that contain one of four amino acid motifs, termed A3/11, Bw4, C1 or C2, in the external-facing α-helices can be ligands for KIR. Polymorphism both within and outside these motifs diversifies the interactions of KIR with HLA and individualize NK cell functions (21, 22). There are two broad forms of *KIR* haplotype, termed *KIR-A* and *KIR-B* (23). *KIR-A* haplotypes have fixed gene content, encoding four inhibitory KIR specific for HLA-A, B or C, as well as KIR2DS4, which is an activating receptor specific for some HLA-A and C allotypes (24). The majority of *KIR2DS4* alleles are not expressed due to a deletion in exon 5 (25). *KIR-B* haplotypes contain a variable number of genes for activating KIR and fewer for inhibitory KIR. The two haplotype groups display distinct functional characteristics. For example, although KIR2DL2 and KIR2DL3 are encoded by the same locus, they differ in strength and specificity and characterize *KIR-B* and *KIR-A* haplotypes, respectively (26). Accordingly, *KIR-A* and *KIR-B* haplotypes also have distinct disease association profiles (27). *KIR-A* haplotypes associate with effective immunity against acute viral infections, but with increased risk of preeclampsia during pregnancy (28, 29). *KIR-B* haplotypes protect women from this pregnancy disorder and improve the outcome of hematopoietic stem cell transplantation (30, 31). Additional to the *KIR-A* and *KIR-B* haplotype designations, specific alleles that can be characteristic to the haplotypes associate with disease susceptibility or protection. A total of 1,110 alleles encoding 543 allotypes have been reported according to the IPD-KIR database release version 2.10.0 (32).

The development of next-generation sequencing (NGS) technologies for genotyping *KIR* and *HLA* at high-resolution has helped identify the substantial variation of KIR allotypes and their interactions with HLA ligands across human populations (33–35). It is therefore of great significance to characterize this combinatorial diversity across all human populations. Although China, as one of the four ancient civilizations of the world, accounts for approximately 20% of the human population, analysis of the *KIR* locus at high-resolution from East Asia is limited to two studies (36, 37). In this study, we fully characterize the allele, haplotype and combinatorial diversity of *KIR* and *HLA class I* in the Zhejiang Han population from the Southeast coast of China (27°02’ to 31°11’ North, 118°01’ to 123°10’ East).

## Materials and Methods

### Samples and DNA Extraction

176 blood samples were collected from healthy blood donors in the Blood Center of Zhejiang province, China. The ethnic background of all individuals is Zhejiang Han. Informed consent was obtained from all participants. The Zhejiang Han are a population of approximately 60 million individuals living on the Southeast coast of China and are a subgroup of the Chinese Southern Han ethnic group. This study was approved by the regional ethics committee, Blood Center of Zhejiang Province. Genomic DNA was extracted using MagNA Pure LC DNA Isolation Kits (Roche Diagnostics, Indianapolis, IN, USA) according to the manufacturer’s instructions. The final DNA concentration was adjusted to 60 ng/μl and optical density at 260/280 was approximately 1.8.

### High-Resolution Sequencing for *KIR* and *HLA*

A biotinylated DNA probe-based capture method was applied to sequence *KIR* and *HLA* genes to high-resolution, as described previously (33). Briefly, 500 ng genomic DNA for each sample was randomly fragmented using enzyme digestion (New England Biolabs, Boston, MA) and then labeled with TruSeq DNA CD indexes (Illumina Inc, San Diego, CA). Fragments of 800–1,200 bp length were obtained by size selection and the individual samples pooled at equal quantity into one tube. Fragments containing target *HLA* and *KIR* genes were captured using specific probes, prepared to a final concentration of 12 pmol/L, as determined by Qubit instrument, then sequenced using a MiSeq instrument and a v3 Reagent Kit (600-cycle; Illumina Inc, San Diego, CA, USA).

### Sequence Data Analysis

*KIR* gene content, copy numbers, and allele genotypes were determined using the Pushing Immunogenetics to the Next Generation pipeline (PING) as previously reported (33). The copy number was determined from the ratio of reads mapping to each *KIR* gene to those mapping to *KIR3DL3*, a reference gene present only one copy on each haplotype. Alleles for 13 *KIR* genes were analyzed, of which *KIR2DL2* and *KIR2DL3* are alleles at the same locus (*KIR2DL2/3*), as are *KIR2DS3* and *KIR2DS5* (*KIR2DS3/5*), and *KIR3DL1* and *KIR3DS1* (*KIR3DL1/S1*). *KIR3DL3* alleles were determined using PHASE 2.1 (38). The *HLA* alleles were determined using TypeStream Visual NGS Analysis Software (TSV2.0, One Lambda Inc, Canoga Park, CA). The presence of KIR ligands, A3/11, Bw4, and C1/C2 was determined according to the *HLA* alleles.

### Frequencies of Alleles, Genotypes, and Haplotypes

*KIR* genotypes were named based on the presence or absence of *KIR* genes according to the allelefrequencies.net database (39). Carrier frequencies of *KIR* genes and genotypes were calculated as their percentage of the total number of individuals. The frequencies of alleles or haplotypes were calculated by direct counting, and the number observed divided by 2N (alleles duplicated on a single haplotype were not included, and absence was counted as a distinct allele). The composition and frequencies of *HLA* haplotypes were determined using Arlequin 3.5.2.2 (40).

### KIR/HLA Interaction Analysis

Interactions between inhibitory KIR and HLA ligands were analyzed. The number of distinct allotype interactions was counted for each KIR for every individual and the mean number was calculated per population. Broadly, KIR2DL1 recognizes C2^+^HLA-C, and KIR2DL2/3 recognizes C1^+^HLA-C, and some C2^+^HLA-C. KIR3DL1 recognizes the Bw4 motif at residues 77–80 of some HLA-A and HLA-B allotypes. KIR3DL2 recognizes the A3/11 motif carried by HLA-A*03 and HLA-A*11 allotypes (41, 42). Major exceptions are KIR2DL1*022, which interacts with C1^+^HLA, and HLA-B*46 and B*73, which carry C1 motifs (20, 26).

### Statistical Analysis

Hardy–Weinberg equilibrium (HWE) was determined for each gene using Fisher’s exact test implemented in the Arlequin software 3.5.2.2. The distributions of genes, alleles, haplotypes, and KIR/HLA interactions in the Zhejiang Han population were compared to other populations as described. Differences between populations were assessed using GraphPad software, by means of the χ^2^ test for categorical variables. The p values were calculated using Fisher’s exact test (pf), applied when appropriate. Bonferroni correction for multiple comparisons was applied. p <0.05 was regarded as significant.

## Results

### High Frequency of *KIR-A* Haplotypes in Zhejiang Han

**Figure 1:**
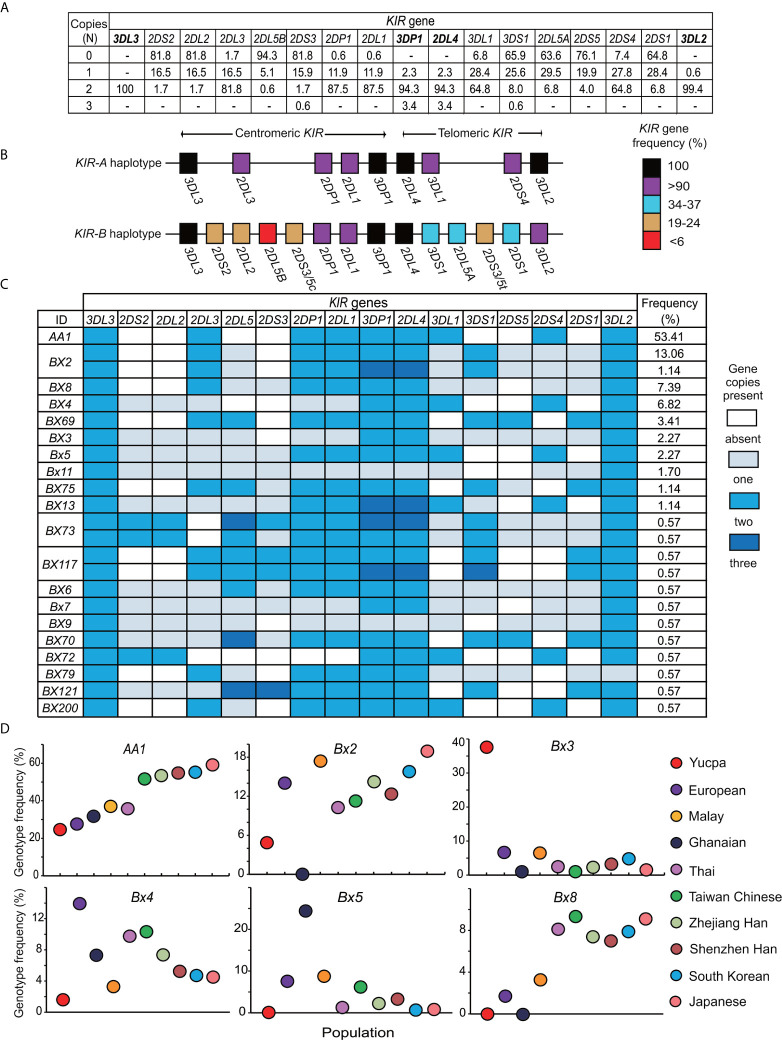
Distribution of genotypes derived from 176 Zhejiang Han individuals. **(A)** Shown for each *KIR* gene is the frequency (%) of individuals carrying the number of copies indicated at the left (0–3). Dash “–” represents not observed. The four framework genes are indicated in bold text. **(B)** Shown is the percentage of *KIR-A* and *KIR-B* haplotypes that carry each of the genes indicated. Shades of red to black represent the percentages, and the key is shown at the right. **(C)** Shown are *KIR* gene content genotypes observed in the Zhejiang Han individuals. The colors of the boxes indicate the gene copy numbers, as given in the key at the right. Genotype IDs based on the presence or absence of *KIR* genes (39) are shown on the left. The frequencies (%) of the corresponding genotypes are listed in the right. **(D)** Shown are the carrier frequencies of *KIR* gene content genotypes *-AA1*, *Bx2*, *Bx3*, *Bx4*, *Bx5*, *Bx8* in ten different populations. They are Chinese Southern Han from Shenzhen (36), Japanese (37), South Korean (43), Taiwan Chinese (44), Malay (45), Thai (46), Europeans (47, 48), Sub-Saharan Africans Ghanaians (49) and South American Yucpa (50). The colored circles represent the populations, as shown at the right.

To analyze the *KIR* locus of the Zhejiang Han at high resolution, we first established which *KIR* genes are present, and in how many copies, for every individual. The four framework genes [*KIR3DL3*, *KIR2DL4*, *KIR3DP1* and *KIR3DL2* (15)] were detected in all 176 individuals of the cohort (**Figure 1A**). *KIR2DL4* and *KIR3DP1* were each observed with one to three copies and one individual in the cohort has only one copy of *KIR3DL2* (**Figure 1A**). Only four individuals have less than two copies of any of these framework genes, six individuals harbor three copies, and none were observed with four copies. These observations indicate that, although structural diversity of the *KIR* locus is present, it is rare in the Zhejiang Han population. *KIR2DL1, KIR2DL3, KIR3DL1, KIR2DS4*, and *KIR2DP1* were all observed at a frequency greater than 90% and at two copies in at least 64.8% of the population (**Figures 1A, B** **)**. These five genes are not present on every *KIR* haplotype, and when they occur together, they characterize the *KIR A* haplotype (15, 51)*. KIR2DL3*, which occurs in the centromeric *KIR* region, showed higher frequency than *KIR3DL1* and *KIR2DS4*, which are located in the telomeric *KIR* region (81.8% vs 64.8%), indicating a higher frequency of centromeric *KIR-A* haplotype motifs (*Cen-A*) than telomeric *KIR-A* motifs (*Tel-A*). Accordingly, the genes characteristic of *KIR-B* haplotypes have frequencies less than 25% in centromeric and approximately 35% in telomeric *KIR* region (**Figure 1B**). We compared the distribution of *KIR* genes of Zhejiang Han with populations representing Asia (37, 43, 46, 52–54), Europe (47, 48, 55, 56), Africa (49, 57), America (50) and Oceania (58, 59) (**Supplementary Table 1**). As a control, this analysis showed the *KIR* gene frequencies to be similar to our previous study of an independent Zhejiang Han cohort that was performed at lower resolution (60). The *KIR* gene frequencies are also similar to those of other populations from East Asia including Japan (37), South Korea (43) and Singapore (53), but distinct from Southeast Asians (46) and populations from other continents (**Supplementary Table 1**). Accordingly, the frequencies of *Cen-B* specific *KIR* genes (*KIR2DS2, KIR2DL2*, *KIR2DL5B*) in the Zhejiang Han are significantly lower than all populations outside East Asia (**Supplementary Table 1**).

As determined by the copy number of each of the 13 *KIR* genes, twenty-three distinct genotypes were observed (**Figure 1C**). Of these genotypes, *KIR-AA* is the most frequent (53.4%) in Zhejiang Han (**Figure 1C**). The *KIR-AA* genotype is common across East Asia, also being observed frequently in other Chinese Southern Han (36), Taiwan Chinese (44), South Korean (43), and Japanese (37). The *KIR-AA* genotype is less frequent outside East Asia, as illustrated by populations representing Southeast Asia, Europe, South America, and sub-Saharan Africa (**Figure 1D**). The other 22 *KIR* genotypes observed in Zhejiang Han are designated *KIR-Bx*, the distributions of which are also similar among East Asians and distinct from populations of other continents (**Figure 1D**). For example, *Bx2*, which is the most frequent *KIR-B* genotype in Zhejiang Han, was not observed in sub-Saharan Africans, whereas *Bx5* is frequent in sub-Saharan Africans, but very rare in Zhejiang Han (**Figure 1D**). Another example is *Bx3*, which was observed with high frequency in Amerindians, but almost absent from all other populations (**Figure 1D**). These examples of distinct genotype profiles characterizing major human population groups illustrate the rapid evolution of the *KIR* locus (27, 61).

### Genes for Inhibitory KIR Exhibit More Allelic Diversity Than Those for Activating KIR

**Figure 2:**
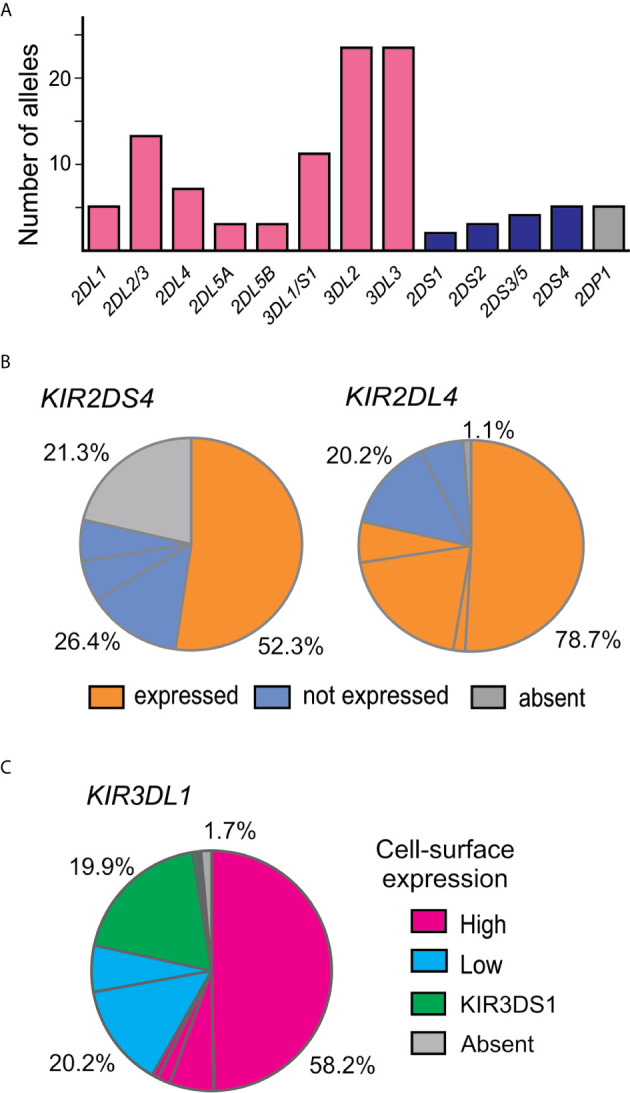
KIR alleles present in the Zhejiang Han population. **(A)** Shown is the total number of *KIR* alleles observed in 176 Zhejiang Han. Genes encoding inhibitory KIR are indicated by red bars, activating by blue bars, and pseudogenes by gray bars. **(B)** Shown are the ratios of expressible and non-expressed alleles of KIR2DS4 and KIR2DL4 in Zhejiang Han. Each pie segment represents a distinct allele, orange indicates allele can be expressed at the cell surface, blue indicates not expressed, grey indicates gene absent. **(C)** Shown is the KIR3DL1 allele expression frequency spectrum in Zhejiang Han. Magenta indicates high expression alleles, cyan—low expression, green—KIR3DS1 (activating receptor) and gray indicates gene absence or unknown expression pattern.

A total of 107 *KIR* alleles were observed in the Zhejiang Han cohort (**Figure 2A** and **Supplementary Table 2**) and no novel alleles were identified. The allele frequency distributions for all the *KIR* genes were in accordance with Hardy–Weinberg equilibrium. The inhibitory KIR are more diverse and polymorphic (88 alleles in total) than the activating KIR (14 alleles). The most diverse gene encoding an activating KIR is *KIR2DS4*, which is the only activating KIR carried by the *KIR-A* haplotype, with five alleles observed (**Figure 2B**). KIR2DS4*00101 is the most frequent allele and is the only one that can encode a functional allotype on the cell surface, whereas the remainder are non-expressed alleles because of a 22bp deletion in exon 5 (25). Other activating KIR are encoded by *KIR-B* haplotypes and show little diversity. *KIR2DS3/5* can be present on either a *Cen-B* or *Tel-B* haplotype; two *KIR2DS3* alleles were observed, one is KIR2DS3*00103 observed in *Cen-B*, and the other is KIR2DS3*00201 observed in *Tel-B.* Although the activating KIR are less polymorphic, their allele frequency distributions do vary across populations. For example in Zhejiang Han, functional KIR2DS4 allotypes are the most frequent, whereas in some populations, such as in Oceania (62), the non-expressed *KIR2DS4* alleles predominate.

**Figure 3:**
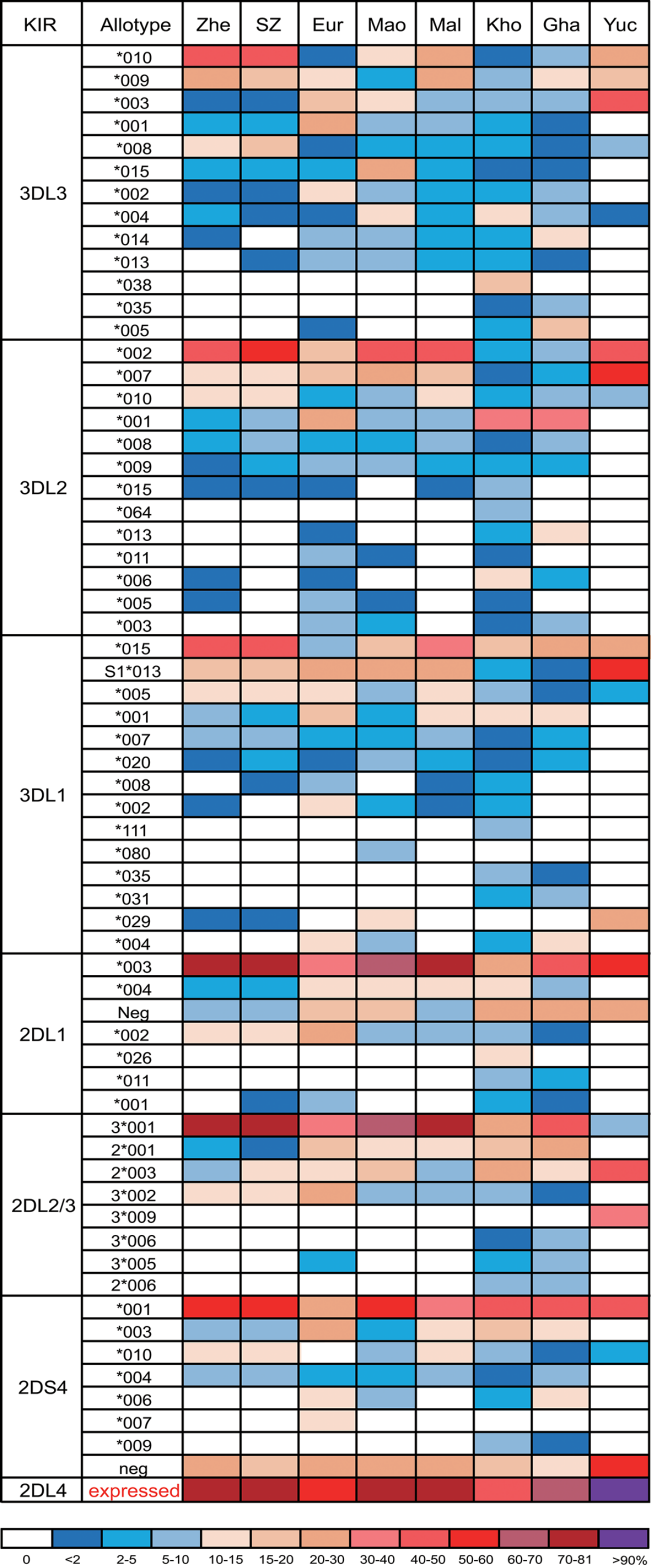
KIR allotype diversity across human populations. Shown are the frequencies of KIR3DL1-3, KIR2DL1-4, and KIR2DS4 allotypes from seven human populations. Shades of dark blue to dark red represent the allotype frequencies from low to high as indicated in the key at the bottom. Zhe, Zhejiang Han; SZ, Shenzhen Han (36); Eur, European (65); Mao, Māori (62); Mal, Malay (45); Kho, Southern Africa Khomani (34); Gha, sub-Saharan Africa Ghanaian (49); Yuc, Amerindian Yucpa (50). For KIR2DL4, the sum of expressible allotypes is shown. * standard WHO nomenclature for KIR alleles.

Among genes encoding inhibitory KIR, those with three domains show greatest variability (25, 32). We observed 23 alleles each of *KIR3DL3* and *KIR3DL2* and 11 alleles of *KIR3DL1/S1*, which encode 16, 17, and 11 allotypes, respectively (**Supplementary Table 2**). Thus, each allele of *KIR3DL1* we observed here (**Figure 2C**) encodes a distinct allotype and the most abundant have high-expressing, high-binding, phenotypes (37, 63, 64). KIR3DL1*015 is the most frequent KIR3DL1 allotype in Zhejiang Han. Comparison with other representative populations shows that KIR3DL1*015 is present worldwide, reaching highest frequency in East Asians (**Figure 3**). By contrast, KIR3DL1*004, which is a poorly expressed phenotype found in Europeans and Africans, was not observed in Zhejiang Han (**Figure 3**) and neither was it observed in another Chinese Southern Han group (36). We previously reported *KIR3DL1* allele frequencies from an independent cohort of Zhejiang Han (66). Because *KIR3DS1* was not analyzed in that cohort, the reported *KIR3DL1* allele frequencies are higher than in the present cohort, however, the frequencies are similar when *KIR3DS1* is considered. The present analysis also showed that *KIR3DL3* has the most extensive cross-population diversity. Here, KIR3DL3*010 clearly characterizes East Asian populations, whereas 3DL3*003 for example characterizes Amerindians, but is rare in East Asians (**Figure 3**).

Although KIR2DL1 is the most polymorphic two-domain KIR, having 173 known alleles [IPD-IMGT/KIR Database version 2.10.0, December 2020 (32)], it has low diversity in Zhejiang Han, with five alleles observed (**Figure 2A**). The most prominent of these alleles is KIR2DL1*00302 at an allele frequency of 77.8% (**Supplementary Table 2**), corresponding with a similar frequency of KIR2DL3*00101. This strong linkage disequilibrium between the two alleles is shared across multiple populations worldwide (34, 37, 45, 50, 62, 65). KIR2DL4, which can be an activating receptor specific for HLA-G (67, 68), has six alleles in Zhejiang Han (**Supplementary Table 2**). Of 107 *KIR2DL4* alleles, encoding 54 distinct polypeptide sequences, 18 do not express a receptor on the cell surface due to a single nucleotide deletion at position 811 in Exon 7 (69). The non-expressed alleles include KIR2DL4*008 and KIR2DL4*011, which were observed at a total frequency of 20.2% in Zhejiang Han (**Figure 2B**). The four expressible *KIR2DL4* alleles observed thus constitute a majority. This majority varies little across human populations, the most extreme being Amerindians, where >95% *KIR2DL4* alleles are expressible, and the exception being KhoeSan, where there is an approximately equal ratio between expressible and non-expressible forms of *KIR2DL4* (**Figure 3**).

### *KIR-A* Haplotypes Are More Frequent Than *KIR-B* Haplotypes Both in Centromeric and Telomeric Regions

**Figure 4:**
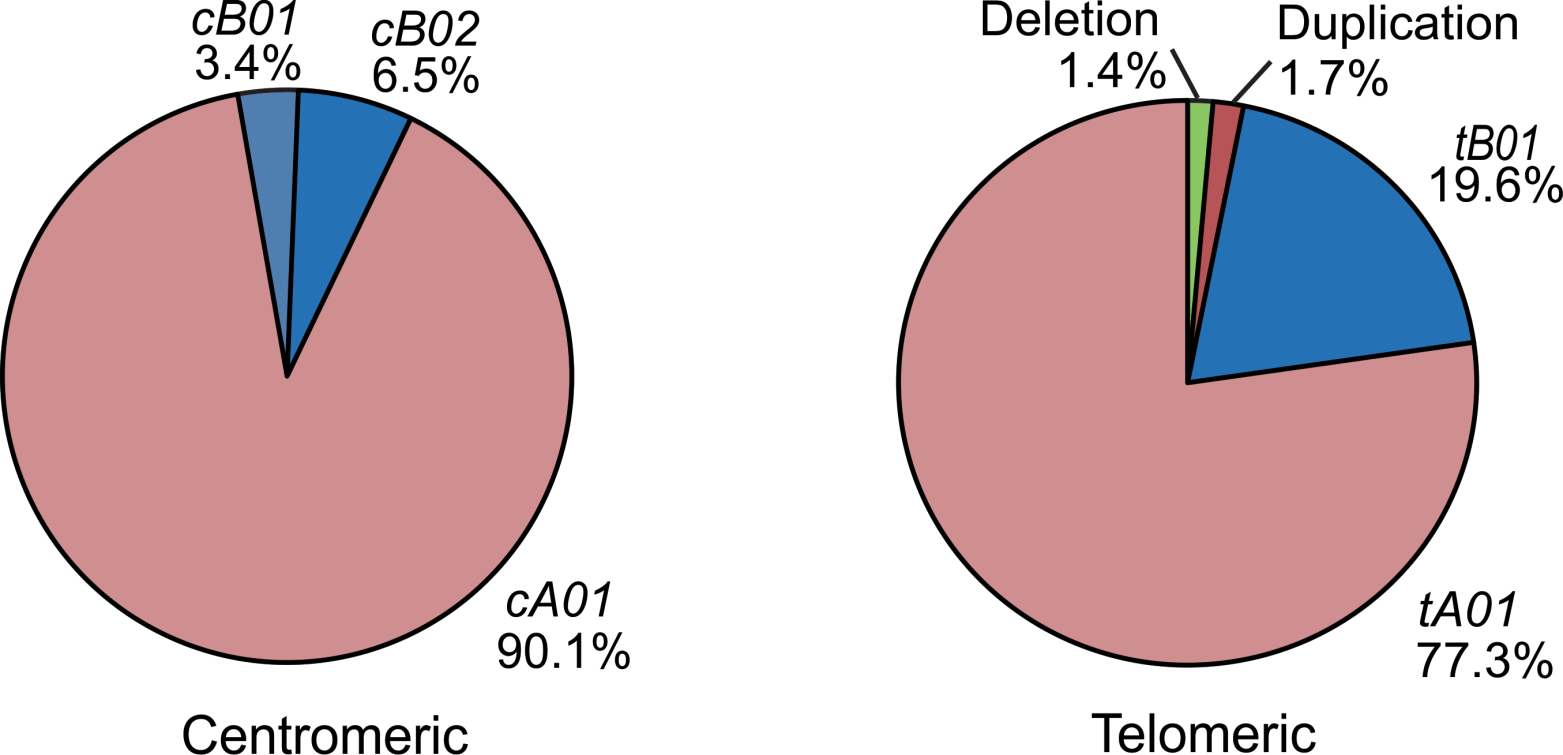
Distribution of *Cen-A/B* and *Tel-A/B* haplotypes in the Zhejiang Han.

**Figure 5:**
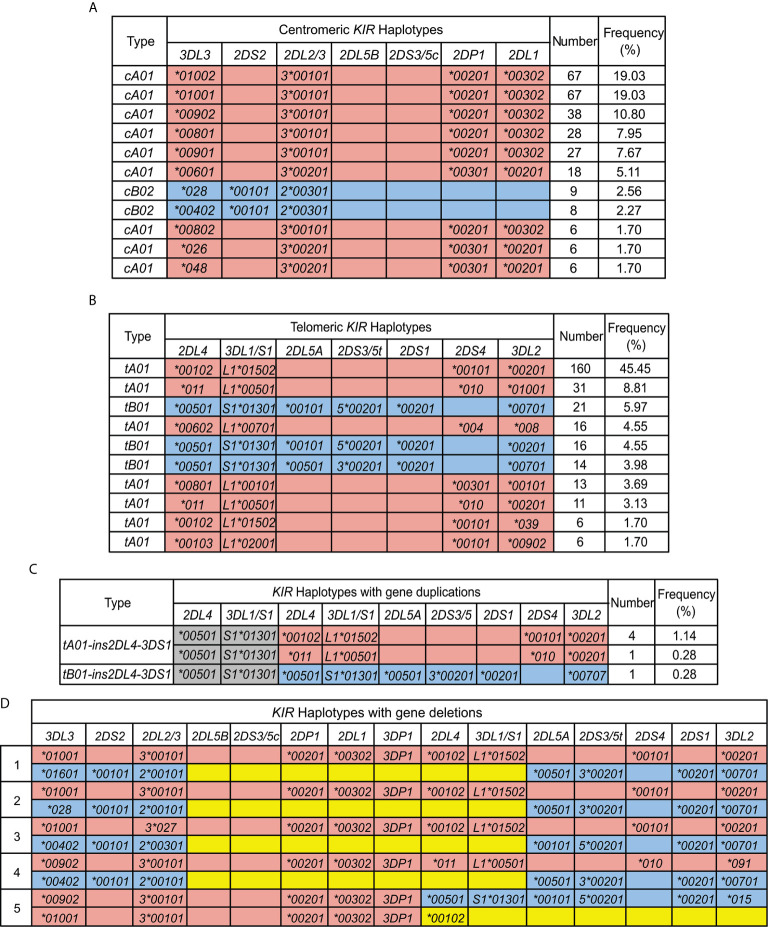
*KIR* haplotypes identified in 176 Zhejiang Han. **(A, B)**. Shown are the ten most frequent centromeric and telomeric haplotypes and their frequencies observed in Zhejiang Han. **(C)** Shown are the extended telomeric haplotypes observed in Zhejiang Han. **(D)** Shown are the two complete haplotypes for each of the five individuals who exhibit contracted *KIR* haplotypes. *KIR-A* haplotype are shaded red and *KIR-B* haplotypes are blue. Gray indicates insertion. Yellow indicates deletion. Blank boxes indicate gene absence. The frequencies of haplotypes are shown at the right. * standard WHO nomenclature for KIR alleles.

Based on the copy number and allele information obtained from high resolution analysis, 54 centromeric haplotypes and 37 telomeric haplotypes were observed in Zhejiang Han (**Supplementary Table 3**). As defined by gene content, three common centromeric haplotype groups, *cA01*, *cB01*, and *cB02*, and two telomeric groups, *tA01* and *tB01*, were observed, as well as rarer forms having duplicated or deleted segments (**Figure 4**). When accounting for allele diversity, there are 41 haplotypes in the *cA01* group (**Supplementary Table 3A**), and 23 in the *tA01* group (**Supplementary Table 3B**). The most frequent *cA01* haplotype at the allele level in Zhejiang Han is KIR3DL3*01002-2DL3*00101-2DP1*00201-2DL1*00302, with a frequency of 19.03% (**Figure 5A**). The five most common *cA01* haplotypes (64.5% in total) differ only in their *KIR3DL3* alleles (**Figure 5A**). The high polymorphism of *KIR3DL3* alleles diversifies the centromeric region, and the number of the centromeric haplotypes would decrease from 54 to 19 if *KIR3DL*3 alleles were excluded (**Supplementary Table 3A**). The most frequent *tA01* haplotype at the allele level in Zhejiang Han is KIR2DL4*00102-3DL1*01502-2DS4*00101-3DL2*00201 (**Figure 5B**), with a frequency of 45.5%. Within the telomeric haplotypes, *KIR2DS3/5t* and *KIR2DL5A* alleles show strong linkage disequilibrium, in which KIR2DS5*00201 and KIR2DL5A*00101 are always found together, as are KIR2DS3*00201 and KIR2DL5A*00501. These patterns of linkage disequilibrium are the same as those found in other populations outside of Africa.

Six individuals having an insertion/duplication of *KIR3DP1-2DL4-3DS1* were observed in Zhejiang Han (**Figure 5C**). The corresponding haplotypes comprised two distinguished at the gene content level, and three as determined by allele content. We also identified four individuals with haplotypes lacking *KIR3DP1-2DL4-3DL1/S1* (**Figure 5D**), with three of these haplotypes (individuals 1, 2 and 4: **Figure 5D**) distinguished only by their alleles of *KIR3DL3*. These haplotypes likely formed by uneven exchange during meiotic recombination, and have also been observed in other populations (17, 45, 70, 71). Although it remains to be confirmed through full haplotype sequence analysis, the insertions/duplications and the deletions occur in approximately the same location 5’ from *KIR3DP1*, suggesting the crossovers can occur frequently through shared mechanisms (72). In addition, we identified a unique haplotype that lacks any *KIR* genes in the telomeric region 3’ from *KIR2DL4*, leaving only six genes on the entire *KIR* haplotype (individual 5; **Figure 5D**). That the allele is KIR2DL4*00102 suggests the parental haplotype that lost these genes was similar to the most frequent *KIR-A* in Zhejiang Han (**Figure 5B**).

### High Frequency of Strong Inhibitory *KIR/HLA* Interactions Identified in Zhejiang Han

**Figure 6:**
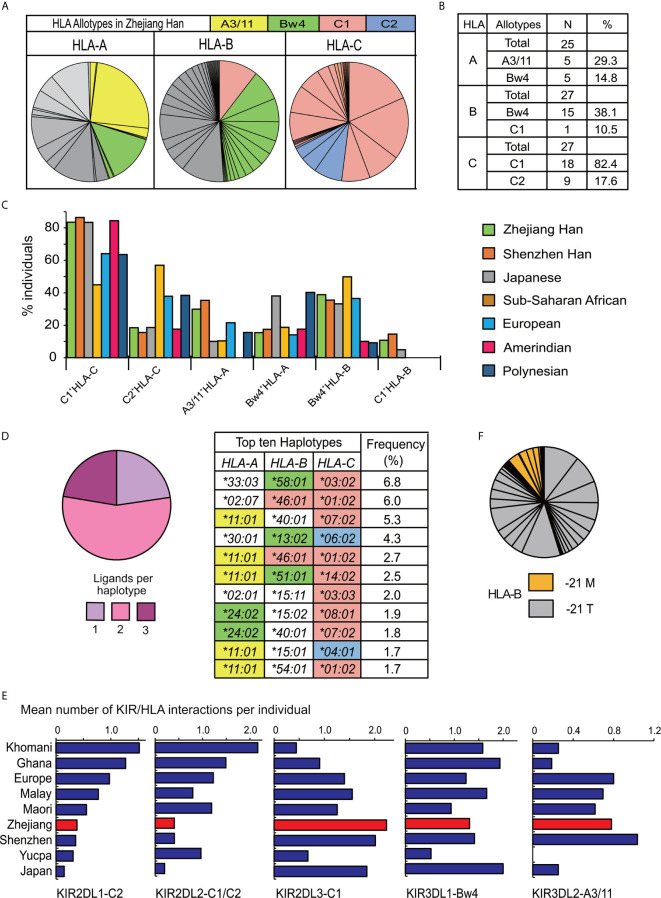
The distribution of HLA in Zhejiang Han. **(A)** Shown are the frequency spectra of *HLA-A*, *-B*, and *-C* allotypes from 176 Zhejiang Han individuals. Each pie segment represents a distinct allotype (HLA allotype frequencies are given in **Supplementary Table 4**), colored segments signify the allotypes that are KIR ligands: Yellow—A3/11 (HLA-A3 and -A11); Green—Bw4; Red—C1; Blue—C2; Gray—allotypes that are not KIR ligands. **(B)** Shown are the numbers of distinct allotypes observed and the combined frequencies of A3/11, Bw4, C1, or C2 allotypes for each HLA class I gene. **(C)** Shown are the distributions of KIR ligands across seven representative populations. The allotype are shown at the bottom. The populations are listed at the right: Chinese Southern Han from Shenzhen, Japanese, South American Yucpa, European, Sub-Saharan African, and Oceania Polynesian (36, 45, 49, 50, 62, 65). **(D)** (Left) Shown are the combined frequencies in Zhejiang Han of *HLA class I* haplotypes encoding one (lilac), two (pink) or three (purple) KIR ligands. (Right) the ten most frequent *HLA class I* haplotypes observed, colored shading indicates *HLA class I* alleles that encode KIR ligands, as described in panel **(A)**. **(E)** Shown is the mean number of distinct interactions per individual observed for each of the inhibitory KIR that interact with polymorphic HLA class I. From left to right: KIR2DL1 interactions with C2^+^HLA-C [or with C1^+^HLA-C for KIR2DL1*022 allotype (20)] KIR2DL2 with C1^+^HLA-B or C, or with C2^+^HLA-C; KIR2DL3 with C1^+^HLA-B or C; KIR3DL1 with Bw4^+^HLA-A or B; KIR3DL2 with HLA-A3/11. The values are shown for nine representative populations: Southern Africa Khomani (34); sub-Saharan Africa Ghanaian (49); European (65); Malay (45); Māori (62); Zhejiang Han; Shenzhen Han (36); Amerindian Yucpa (50); Japanese (37). **(F)** Shown is the frequency of *HLA-B* alleles encoding methionine (orange) or threonine (gray) at position -21 in the leader peptide. Each pie segment represents a distinct HLA-B allotype (HLA allotype frequencies are given in **Supplementary Table 4**).

Alleles for highly polymorphic HLA-class I (*HLA-A*, *-B*, *-C*) were obtained from high resolution sequencing. A total of 25 *HLA-A* alleles, 43 *HLA-B* alleles, and 27 *HLA-C* alleles were observed (**Supplementary Table 4**), and the distributions for all the *HLA* genes complied with Hardy–Weinberg equilibrium. Only some HLA-class I allotypes act as ligands for KIR (9, 27). The two most frequent alleles of *HLA-A*, *-B*, *-C* respectively in Zhejiang Han are A*11:01 (25.3%) and A*24:02 (13.1%), B*40:01 (11.6%) and B*46:01 (10.5%), C*07:02 (18.2%) and C*01:02 (16.8%). All the allotypes they encode can interact with KIR, except for B*40:01. Every HLA-C allotype is a ligand for *KIR*, and in total, 44.1% of HLA-A and 48.6% of HLA-B allotypes by frequency in the Zhejiang Han can act as KIR ligands (**Figures 6A, B**). Most of the HLA-C allotypes (82.4%) contain a C1 motif (C1^+^HLA-C) in Zhejiang Han and HLA-B*46:01, which also carries a C1 motif was also observed at high frequency (10.5%). HLA-B*46:01 is one of only two HLA-B allotypes that carry a C1 motif (C1^+^HLA-B), the other is HLA-B*73:01, and both have geographical distributions focused in Asia, B*46:01 in Southeast Asia and B*73:01 in West Asia (39). C1^+^HLA-C and C1^+^HLA-B were both observed at higher frequencies than non-Asian populations (**Figure 6C**). Accordingly, HLA-C2 shows extremely low frequency in Zhejiang Han, and significantly lower than other populations (**Figure 6C**, **Supplementary Table 5**). Moreover, A3/11, specifically encoded by A*11:01, was observed at higher frequency in Zhejiang Han than any other populations. We identified 166 *HLA* haplotypes (**Supplementary Table 4**). Most of the observed haplotypes (77.3%) encode more than two KIR ligands, including A*11:01, which was detected in five of the ten most frequent haplotypes (**Figure 6D**). These findings are similar to those recently reported for Chinese Southern Han from Shenzhen (36).

The high frequency of the *KIR-A* haplotype, which encodes four inhibitory receptors able to interact with HLA class I ligands (KIR2DL3, KIR2DL1, KIR3DL1 and KIR3DL2), indicates inhibitory KIR-HLA interactions predominate in Zhejiang Han. Here, KIR2DL1*00302, KIR2DL3*00101, KIR3DL1*01502 and KIR3DL2*00201 are the major alleles carried by the *KIR-A* haplotype, and all the allotypes they encode have strong interactions with their HLA ligands compared to other allotypes (20, 37, 41, 73, 74). Because of the high frequency of *KIR-A*, Zhejiang Han are rich in *KIR-AA* genotypes, and each of these individuals therefore has eight strong inhibitory KIR allotypes that can interact with HLA. The mean number of inhibitory KIR-HLA pairs per individual was analyzed, revealing that the mean interaction of KIR2DL3-C1^+^HLA per individual (2.2) is higher than other populations (**Figure 6E**). This high frequency is caused by the high frequencies of KIR2DL3 and C1^+^HLA-C, and the extra C1^+^HLA-B allotype present (**Figure 6A**). By contrast, due to the low frequency of the C2^+^HLA-C allotypes, the frequency of interactions with C2^+^HLA is low in Zhejiang Han (**Figure 6E**). In these regards the Zhejiang Han also show very similar repertoires to the Shenzhen Chinese Southern Han (36). Further diversifying NK cell interactions with polymorphic HLA, conserved HLA-E can present peptides derived from HLA-A, C and some B allotypes. The peptides are derived from allotypes possessing methionine at the second residue of the leader sequence (-21M). HLA-E is recognized by inhibitory CD94:NKG2A expressed by NK cell and T cell subsets (75, 76). In Zhejiang Han, the total frequency of *HLA-B* alleles encoding -21M is 11.6% (**Figure 6F**). Among them, only HLA-B*38 also encodes a Bw4 ligand for KIR and is the most frequent at 3.7%.

## Discussion

Specific genetic variants of KIR, HLA, and their combinations are associated with susceptibility and progression of multiple immune-mediated diseases, including viral infections, autoimmunity, cancer and reproductive disorders (10, 11, 13). Critical to understanding these disease mechanisms are systematic analyses of KIR and HLA allotype diversity across defined human populations. In this study, we used a targeted sequencing method to comprehensively characterize all *KIR* and *HLA class I* alleles at high resolution for the Zhejiang Han, who represent the Chinese Southern Han on the Southeastern coast of China. From 176 healthy individuals we identified 23 *KIR* gene-content genotypes, 107 *KIR* alleles, 54 centromeric and 37 telomeric *KIR* haplotypes, and 11 individuals having evidence for large scale gene rearrangements. We also observed 95 HLA class I allotypes, encoded by 166 distinct *HLA-A-B-C* haplotypes. We show that the Zhejiang Han are characterized by a predominance of interactions of strongly inhibitory KIR with HLA class I, and particularly KIR2DL3 with C1^+^HLA, supporting the observation that these inhibitory interactions are significantly enhanced in East Asians (36). Because strongly-inhibiting KIR enable maturation of NK cells with strong effector functions (37, 77), one expected consequence is that NK cell responses are enhanced in East Asians relative to other populations.

As observed in other East Asian populations (39), we identified a high frequency of *KIR-A* haplotypes in Zhejiang Han, where more than 90% of centromeric and 80% telomeric haplotypes have the *KIR-A* configuration. Previous whole-genome, as well as analyses focused on the *KIR* genomic region, have shown that the centromeric *KIR-A* haplotypes are subject to positive natural selection specifically in East Asians (37, 78, 79). By contrast, other studies continue to identify a balance between *KIR-A* and *KIR-B* haplotypes across populations worldwide, likely due to competing influences from infection control and reproduction (27, 80). Another outcome of selection favoring *KIR-A* in East Asia is the low frequency of *KIR-B* haplotypes, specifically those that contain *KIR2DL5B* and *KIR2DS3*. In Amerindians, a complete loss of *KIR2DS3* is observed (50), and this may be tolerated because the common 2DS3*00102 allele does not form a functional receptor (81). In addition to gene content, *KIR* haplotypes characteristic to human populations are distinguished by the alleles they carry (34, 45, 50, 82). Characteristic alleles of Zhejiang Han *KIR-A* haplotypes include KIR2DL1*003, KIR3DL1*015, and KIR3DL2*002, which all encode strongly inhibiting KIR allotypes, as defined by their ligand binding strength and signal transduction abilities. By contrast the *KIR-B* haplotypes carry fewer inhibitory receptors and they tend to be allotypes having weak affinity or signal transduction, including KIR2DL1*00401 and KIR3DL2*00701 (41, 74). Thus, even in heterozygous individuals we may expect the functional signature of the *KIR-A* encoded receptors to dominate. The influence of *KIR-B* haplotypes being further diminished by the very low frequency of *KIR-B* homozygous genotypes in this population.

HLA-C evolved to be the dominant KIR ligand in humans (27). Chinese Han have a high frequency of HLA-B*46:01, which is only common in Asia and formed by recombination between HLA-B*15:01 and HLA-C*01:02 (83). HLA-B*46:01 encodes an exceptional HLA-B allotype that can react with KIR2DL2/3, due to possession of a C1 ligand (26), and has risen to high frequency in this geographic region through admixture and natural selection (36, 84). Candidates for this selection include endemic bacterial or viral infections (10, 29, 85–89). An alternative explanation for selection acting on B*46 is that it provides a KIR ligand, whereas B*15:01, from which B*46:01 is derived, does not carry a KIR ligand (36). Co-evolution of KIR with HLA has been driven under natural selection, leading to frequency variation across populations for specific KIR–HLA interactions (49, 50, 90). Our analysis identified many similarities in immunogenetic diversity between the Zhejiang Han and the closely related Shenzhen Han, from Southern China (36). Distinguishing the Zhejiang Han is a high frequency of *KIR-A* and *C1^+^HLA-C*, which is a genetic combination associated with preeclampsia (28). This serious multisystem disorder of pregnancy is most frequent in women homozygous both for *KIR-A* and *C1^+^HLA-C*, if the fetus carries C2^+^HLA-C (28). However, there is a substantially lower incidence of preeclampsia in China compared with western countries (91–93), which is likely due to the lower frequency C2^+^ ligands. Moreover, HLA-G expression by extravillous trophoblasts, which promotes vascular remodeling, may help ensure successful placentation thus preventing preeclampsia (94). HLA-G is a ligand for KIR2DL4 (67), and in Zhejiang Han the majority of *KIR2DL4* alleles express a functional allotype.

Uneven exchange during recombination events has been a frequent occurrence during *KIR* gene evolution, creating or amplifying structural diversity of the locus (25, 95). Although our previous analysis of an independent cohort from Zhejiang Han identified similar frequencies of individual *KIR* genes (60), it was not previously possible to detect any additional structural diversity. In the present analysis of Zhejiang Han, which accounted for gene copy number in addition to gene presence, haplotypes carrying a *KIR3DP1-2DL4-3DL1/S1* insertion were observed in six individuals. Acquisition of full sequence data also permitted inference of the allele composition of these haplotypes. Similar haplotypes to those characterized here have been identified in European, Oceanian and other Asian populations, but are very rare in Africans (17). We identified a unique contracted haplotype lacking the telomeric segment, retaining only *KIR2DL4* as the 3’ terminal gene. The capture probes we used can tolerate up to 20% sequence divergence (33) and their high density in the target region would imply that we did not miss some highly divergent sequences from this individual. That the allele is KIR2DL4*00102, indicates that this contracted haplotype was formed from the most frequent *KIR-A* haplotype observed in the Zhejiang Han. As these recombination events most likely occur during meiosis, it remains unlikely that we will observe the reciprocal haplotype formed during that recombination event. That the more frequent structurally divergent haplotypes we observed are all characterized by recombination in approximately similar locations to each other, therefore suggests similar mechanisms or additional recombination hotspots abound in the *KIR* locus.

In conclusion, characterizing combinatorial diversity of *KIR* and *HLA* at high resolution in discrete populations is an important step toward understanding the roles of their polymorphism in disease and in human evolution. The mean number and diversity of KIR and HLA interactions in Zhejiang Han is similar to that of other East Asians, yet highly distinct from African, European and Oceanic populations. These findings will be important for analyzing diseases with susceptibilities that vary across populations and will form a baseline for individualizing immune-based therapies, including transplantation, in Southeastern China.

## Data Availability Statement

The original contributions presented in the study are included in the article/**Supplementary Material**, further inquiries can be directed to the corresponding authors.

## Ethics Statement

The studies involving human participants were reviewed and approved by Blood Center of Zhejiang Province. The patients/participants provided their written informed consent to participate in this study.

## Author Contributions

ST and FZ designed experiments. ST and JW performed lab experiments. YH, KK, PN, and ST analyzed data. PN provided the technology and probes. ST, PN and FZ wrote the paper. All authors contributed to the article and approved the submitted version.

## Funding

This work was sponsored by the Science Research Foundation of Zhejiang Province (LY18H080002) and Science Research Foundation of Zhejiang Healthy Bureau (2018RC003, 2019RC031). PN was supported by National Institutes of Health of the USA (R56 AI151549 and R01 AI128775).

## Conflict of Interest

The authors declare that the research was conducted in the absence of any commercial or financial relationships that could be construed as a potential conflict of interest.

## Supplementary Material

Supplementary Table 3 Distribution of **(A)** centromeric and **(B)** telomeric *KIR* haplotypes in 176 Zhejiang Han individuals. KIR A haplotypes are shaded red and KIR B haplotypes shaded blue.

Supplementary Table 4 Distribution of **(A)** *HLA-A*, *-B*, *-C* alleles and **(B)** *HLA-A-B-C* haplotypes in 176 Zhejiang Han individuals. KIR ligands are colored according to **Figure 6A**, and those expressing -21M are indicated with orange shading.
